# Severe Alcohol Use Disorder in a Patient Who Is Deaf, Mute, and Having Limited Literacy: A Case Report and Review of Disability-Linked Substance Use Vulnerabilities

**DOI:** 10.7759/cureus.98401

**Published:** 2025-12-03

**Authors:** Sophie A Qui, Olufemi Ogundeji

**Affiliations:** 1 Medicine, Edward Via College of Osteopathic Medicine, Monroe, USA; 2 Chemical Dependency Unit, Brentwood Hospital, Shreveport, USA

**Keywords:** alcohol use disorder, communication barriers, deafness, disability, health-care equity, naltrexone, substance use disorder

## Abstract

Alcohol use disorder (AUD) in individuals with communication and literacy disabilities is poorly recognized and rarely described in the literature. Standard diagnostic and withdrawal management tools are not adapted for deaf, mute, and illiterate patients, creating gaps in equitable care.

A 64-year-old man who was congenitally deaf, mute, and with limited literacy presented for protective-custody alcohol detoxification after decades of consuming 12-30 beers daily. Bereavement and loss of his primary caregiver triggered an escalation of use. The Clinical Institute Withdrawal Assessment for Alcohol (CIWA-Ar) scoring was not feasible due to communication barriers. Management included a lorazepam taper, naltrexone initiation, mirtazapine for mood symptoms, and osteopathic manipulative treatment for somatic complaints. Interpreter-facilitated therapy and family involvement were central to stabilization.

This case highlights the need for culturally and linguistically adapted withdrawal tools, early interpreter involvement, and disability-informed addiction treatment. To our knowledge, this is among the first reports describing detoxification of a deaf, mute, and illiterate patient with severe AUD, underscoring the urgency of inclusive clinical protocols.

## Introduction

Alcohol use disorder (AUD) is a chronic, relapsing condition characterized by impaired control over alcohol use, compulsive drinking, continued use despite adverse consequences, and physiological dependence [1]. According to the Diagnostic and Statistical Manual of Mental Disorders, Fifth Edition (DSM‑5), AUD is diagnosed when an individual meets at least two of 11 criteria within a 12‑month period [1]. These criteria include: consuming alcohol in larger amounts or over longer periods than intended; persistent desire or unsuccessful efforts to cut down; significant time spent obtaining, using, or recovering from alcohol; craving; recurrent use resulting in failure to fulfill major role obligations; continued use despite social or interpersonal problems; giving up important activities; recurrent use in hazardous situations; continued use despite physical or psychological problems; tolerance; and withdrawal [1]. AUD is one of the most prevalent behavioral health conditions globally, contributing to significant morbidity, mortality, and psychosocial impairment [2]. Within marginalized populations, such as individuals who are deaf or hard of hearing (D/HH), the burden of alcohol use is compounded by unique cultural, linguistic, and systemic barriers to prevention and treatment. Epidemiologic studies demonstrate that while the prevalence of lifetime alcohol use among deaf adults may not substantially differ from that of hearing adults, deaf individuals are disproportionately represented among heavy drinkers and are more likely to engage in high‑risk alcohol consumption patterns [2,3].

The patient in this case - a 64-year-old deaf, mute, and illiterate male with severe AUD - highlights the intersection of disability, chronic substance misuse, and systemic neglect. He presented with daily alcohol intake ranging from 12 to 30 beers, long-standing alcohol-related medical complications (including pancreatitis and hypertension), and acute withdrawal symptoms on admission. His psychosocial history underscores the impact of enabling familial relationships, repeated displacement, and impaired adaptive functioning following the death of his sister, his primary caregiver. Such contextual factors are consistent with findings that deaf individuals are at elevated risk of substance misuse due to isolation, family dysfunction, unemployment, and cumulative trauma [4,5].

Emerging research underscores that D/HH individuals face disproportionate exposure to negative life events - such as abuse, discrimination, unemployment, and marginalization - that increase vulnerability to psychiatric comorbidities and maladaptive coping strategies like substance use [4]. For example, studies demonstrate significantly higher rates of depression, anxiety, and trauma among deaf individuals, each of which is independently associated with increased risk of AUD [6,5]. Moreover, deaf culture and communication differences can complicate help-seeking behaviors, with many patients reporting stigma, mistrust of hearing providers, and limited access to linguistically and culturally appropriate care [7].

The assessment and treatment of AUD in deaf patients requires specialized consideration. Traditional tools such as the Clinical Institute Withdrawal Assessment for Alcohol (CIWA-Ar), which consists of 10 items covering nausea/vomiting, tremor, sweats, anxiety, agitation, perceptual disturbances, headache, and orientation, rely heavily on verbal communication, making them poorly adapted for deaf, non-literate individuals [8,9]. Additionally, low health literacy, particularly in patients with limited exposure to American Sign Language (ASL) or written English, often results in profound gaps in knowledge about the risks of substance use and the availability of treatment [2]. This is further compounded by the limited availability of interpreters trained in addiction medicine and the scarcity of treatment programs tailored to deaf patients [10]. Innovative approaches, including e-therapy and specialized chemical dependency programs for deaf individuals, have been proposed as partial solutions to address these barriers [10].

Recent national surveys confirm that alcohol remains the most frequently misused substance in the deaf community, with some reports suggesting prevalence rates of heavy drinking and AUD three times higher than those observed in hearing populations [2,3]. Despite this, deaf individuals are less likely to seek or receive treatment, and when they do, they often encounter providers unprepared to accommodate their linguistic and cultural needs [7]. These systemic barriers perpetuate health disparities, leaving many deaf patients to cycle through untreated addiction, medical complications, and psychosocial instability.

This case, therefore, provides an opportunity to explore the clinical, ethical, and systemic challenges inherent in managing severe AUD in a patient who is deaf, mute, and illiterate. It emphasizes the need for culturally responsive assessment tools, improved health literacy outreach, and integrated treatment models that address the unique vulnerabilities of this underserved population.

## Case presentation

Patient information

A 64-year-old male patient who was congenitally deaf, mute, and with limited literacy was admitted to the Chemical Dependency Unit at Brentwood Hospital under a Physician’s Emergency Certificate for alcohol detoxification. He complained of increased anxiety, diaphoresis, and tremors that began the night before. He was unemployed, received disability benefits, and had lived with his sister until her recent death. His niece, who has since assumed responsibility as his primary collateral informant, described a long-standing history of dependence on his late sister, limited independent living skills, and repeated episodes of displacement from his late sister's home following bereavement.

History

The patient began consuming alcohol at an approximate age of 15 years and has engaged in daily heavy use ever since, with current intake estimated at 12-30 beers per day. He also reported intermittent marijuana use, smoking around two or three blunts on weekends. His alcohol misuse has resulted in multiple medical complications, including pancreatitis, hypertension, gastroesophageal reflux disease, and recurrent bronchitis. Per his niece, the only medication he took daily was amlodipine for his hypertension and a PRN (pro re nata) albuterol inhaler for his bronchitis. Additionally, he described a history the week prior to admission of riding his bicycle while intoxicated, leading to an injury to his left wrist. Due to his communication barrier, he was not able to seek care for his injured wrist prior to admission at Brentwood Hospital.

He denied prior psychiatric diagnoses, psychiatric hospitalizations, or suicide attempts. However, his niece reported episodes of verbal and physical aggression during intoxication, with threats directed at family members. He would show this through acts of shouting incomprehensible sounds, throwing objects and furniture, and throwing his fists up at members of his family.

The patient was born deaf and is unable to speak; his literacy is limited, and he demonstrates only minimal knowledge of ASL. He completed his education at the Texas School for the Deaf through grade 12 but did not pursue further vocational or academic training. Despite completing education through grade 12, he reported limited engagement with academic tasks, and his home environment did not foster independent learning; consequently, he was unable to achieve full literacy. He was unemployed and currently receiving disability benefits as his sole source of income. He has been unable to maintain employment due to significant communication barriers and, until her passing, relied on financial support from his sister in addition to disability benefits. He has no spouse or children and previously resided with his sister, who enabled his alcohol use and assumed responsibility for his daily needs until her death. Since then, his niece has attempted to relocate him to her home, though he has repeatedly run away from her due to her restrictions on drinking.

The patient’s family psychiatric history was significant for multiple relatives with alcohol use disorders. He denied a personal history of physical, emotional, or sexual abuse. His social history is significant for many years of alcohol abuse. His legal history includes multiple arrests related to alcohol intoxication. Developmental history is notable for congenital deafness.

Clinical findings

**Figure 1:**
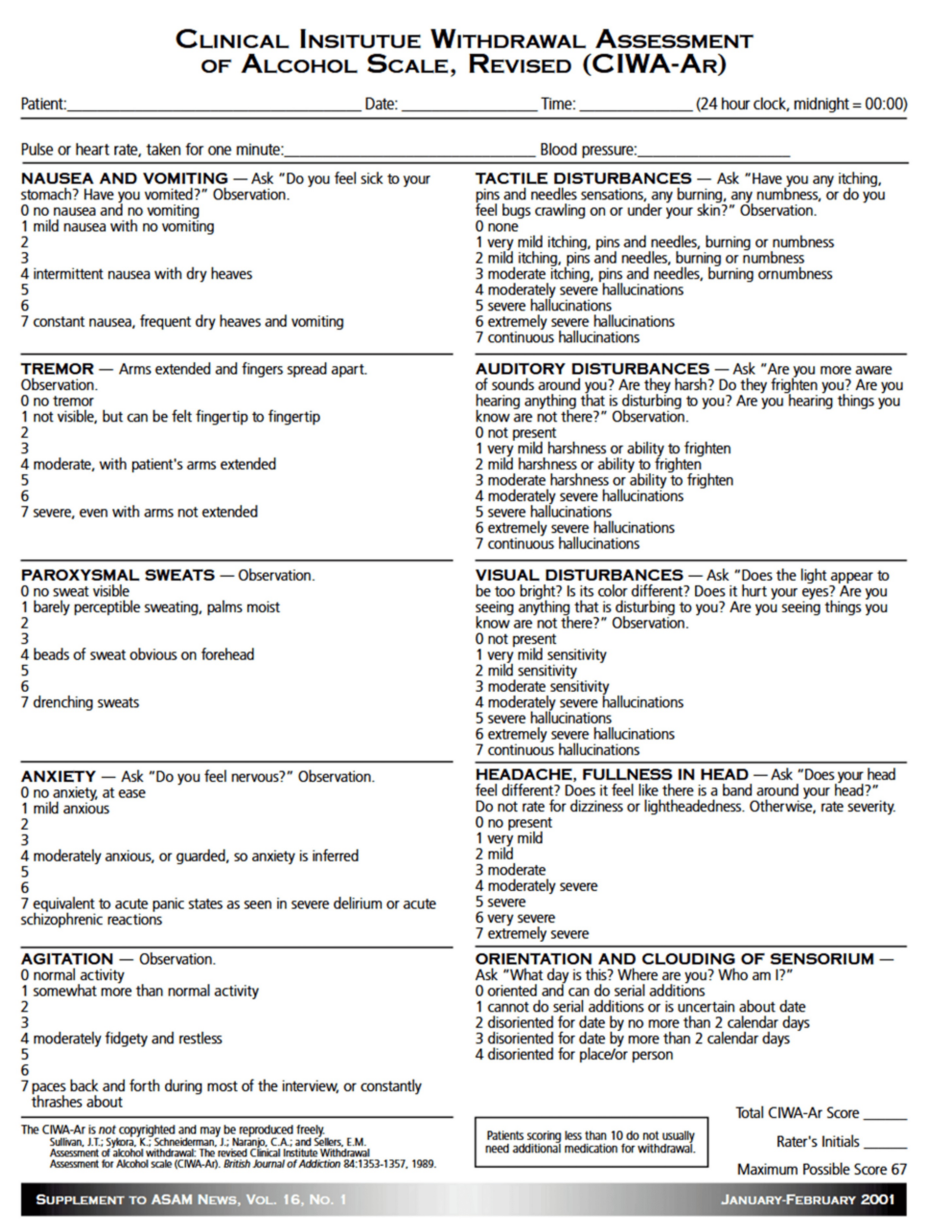
Clinical Institute Withdrawal Assessment for Alcohol Scale, Revised (CIWA-Ar) The CIWA-Ar is a validated tool used to quantify the severity of alcohol withdrawal symptoms and to guide benzodiazepine administration during detoxification. Scores help determine the intensity of withdrawal and the need for pharmacologic intervention.

On admission, an ASL interpreter was not available; therefore, the assessment was conducted through visual observation and collateral information provided by his niece. At that time, the patient appeared anxious, diaphoretic, and tremulous. Vital signs demonstrated tachycardia with otherwise stable parameters. Abdominal examination was benign, and neurologic examination showed no focal deficits or signs of delirium tremens. No hallucinations were observed. Mental status evaluation was limited due to communication barriers; however, the patient demonstrated cooperative behavior, appropriate affect, and conveyed a subjectively “very happy” mood via ASL. Insight and judgment were assessed as limited. A Clinical Institute Withdrawal Assessment for Alcohol (CIWA‑Ar) score could not be reliably obtained because an ASL interpreter was unavailable at the time of admission (see Figure 1 for CIWA-Ar form).

Diagnostic assessment

**Figure 2:**
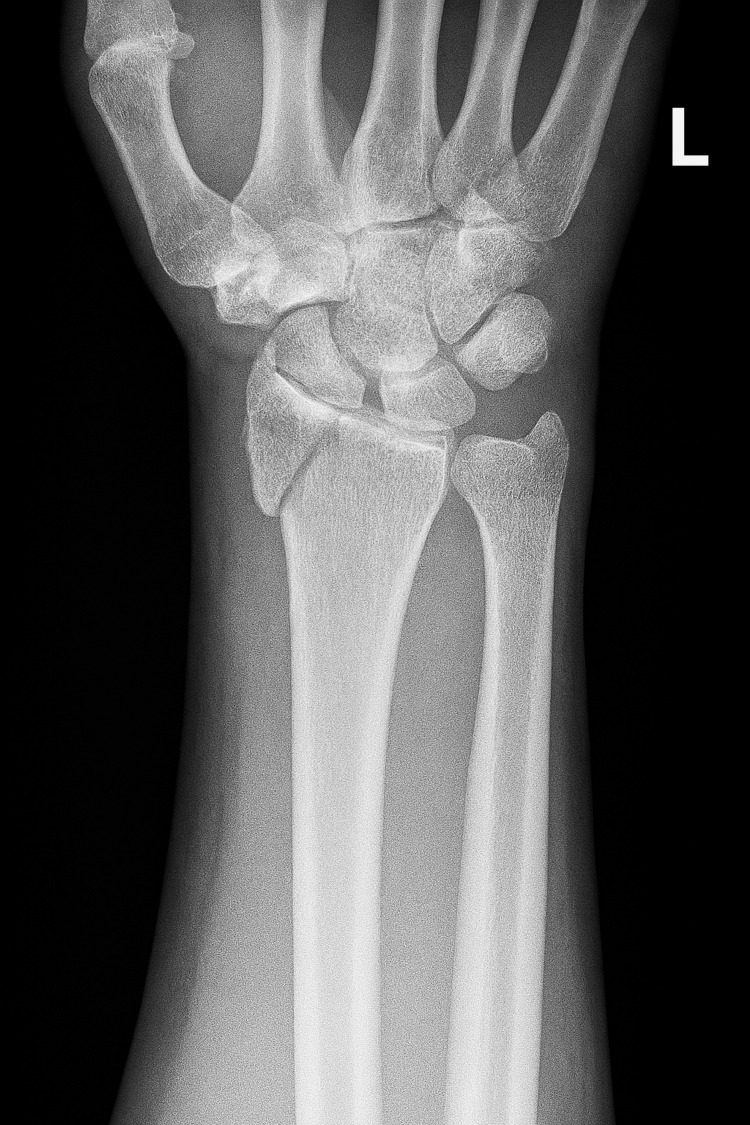
Radiograph showing a mildy displaced acute-to-subacute left intra-articular fracture at the distal radiocarpal joint

A complete blood count (CBC), comprehensive metabolic panel (CMP), gamma‑glutamyl transferase (GGT), lipase, lipid panel, and thyroid function panel were obtained. Laboratory evaluation revealed aspartate aminotransferase (AST) of 43 U/L, thrombocytopenia with platelet count of 65×10³/µL, and low-density lipoprotein (LDL) cholesterol of 102 mg/dL, with the remainder of the metabolic panel within reference limits (see Table 1 for comparison to normal values). A left wrist radiograph showed a mildly displaced acute-to-subacute intra-articular fracture involving the radiocarpal joint (see Figure 2 for radiographic image).

**Table 1:** Laboratory Findings LDL: Low-density lipoprotein.

Parameter	Result	Reference Range	Interpretation
AST	43 U/L	10-40 U/L	Mildly elevated, consistent with hepatic stress from chronic alcohol use
Platelet count	65×10³/µL	150-400 ×10³/µL	Thrombocytopenia, likely secondary to alcohol-related marrow suppression
LDL cholesterol	102 mg/dL	<130 mg/dL	Within normal limits

The differential diagnosis included severe alcohol use disorder with withdrawal, unspecified mood disorder related to grief and substance use, generalized anxiety disorder, cognitive impairment due to chronic alcohol exposure, and possible post-traumatic stress disorder. On admission, the patient exhibited anxiety, diaphoresis, and tremulousness, consistent with acute alcohol withdrawal. Collateral history revealed long-standing heavy alcohol use, raising concern for severe alcohol use disorder with withdrawal. His affect was noted to be anxious and dysphoric, with reports of persistent grief following the death of his sister, suggesting the possibility of an unspecified mood disorder related to bereavement and substance use. The patient’s restlessness, heightened autonomic arousal, and pervasive worry were also compatible with generalized anxiety disorder. In addition, his limited literacy, communication barriers, and history of chronic alcohol exposure raised suspicion for cognitive impairment secondary to alcohol-related neurotoxicity. Finally, collateral information suggested exposure to traumatic experiences and ongoing hypervigilance, which warranted consideration of post-traumatic stress disorder.

Therapeutic intervention

The patient was admitted for detoxification and stabilization under seizure, withdrawal, and violence precautions. Pharmacologic management included a lorazepam taper for alcohol withdrawal, initiated at 2 mg every six hours with gradual reduction over several days, thiamine 100 mg daily and folate 1 mg daily for nutritional supplementation, resumption of home amlodipine 10 mg once daily for hypertension, initiation of mirtazapine 7.5 mg nightly for mood symptoms, and naltrexone 50 mg once daily for craving reduction. Nonpharmacologic interventions were implemented to improve social determinants of health and overall quality of life. These included sign‑language-facilitated group therapy, held twice daily, to enhance communication access and peer support, as well as daily family therapy sessions to strengthen social connectedness and caregiver involvement. An orthopedic consultation was obtained for fracture management. Treatment included immobilization of the wrist with a splint and administration of ibuprofen as needed for pain control.

Follow-up and outcomes

The patient tolerated detoxification without complications, demonstrated improved mood, and expressed motivation for sobriety. He actively engaged with group therapy through sign-language interpretation and tolerated naltrexone without adverse effects. He was discharged 10 days later in a stable condition with arrangements for outpatient psychiatric care, primary medical follow-up, and support for alcohol abstinence.

By the time of discharge, the patient was clinically stable. His anxiety, diaphoresis, and tremulousness had resolved following detoxification, and his mood symptoms showed marked improvement with initiation of mirtazapine. He expressed sustained motivation for sobriety and demonstrated consistent engagement in sign‑language-facilitated group therapy. Cognitive limitations related to chronic alcohol exposure persisted, representing the primary symptom that had not fully improved. His wrist fracture had been managed with immobilization in a splint cast and analgesia, and pain was adequately controlled.

After discharge, he was instructed to continue with sign‑language-facilitated group therapy twice weekly and family therapy once weekly, alongside weekly outpatient psychiatric care. His medication regimen included thiamine 100 mg daily, folate 1 mg daily, amlodipine 10 mg once daily for hypertension, mirtazapine 7.5 mg nightly for mood symptoms, and naltrexone 50 mg once daily for craving reduction. Structured outpatient support for alcohol abstinence was arranged, incorporating therapy, family engagement, pharmacologic treatment, and referral to community resources accessible to individuals with hearing impairment.

Informed consent

Written informed consent for publication of this case report was obtained from the patient. The details of the report, including medical, psychiatric, and social history, were explained to the patient in the presence of a certified ASL interpreter. The primary author (Sophie Qui) reviewed the consent form with the patient, who confirmed understanding and provided written consent.

## Discussion

This case illustrates the complex intersection of addiction, disability, and systemic neglect, emphasizing the challenges of managing AUD in individuals with congenital deafness, mutism, and functional illiteracy. These overlapping disabilities created profound barriers to assessment and treatment, rendering standardized detoxification tools such as CIWA-Ar, effectively unusable. Despite the prevalence of alcohol misuse within the deaf and hard-of-hearing (D/HH) community - reported at rates comparable to or higher than hearing populations [2,3] - there remains a striking lack of literature describing safe and effective management strategies for patients who are both nonverbal and unable to read or write. To our knowledge, no published reports specifically document the compounded challenge of coexisting mutism and illiteracy in the context of alcohol withdrawal.

Clinical assessment in this population requires substantial modification. Traditional withdrawal scales depend on patient-reported symptoms such as anxiety, tremor, or nausea, which cannot be communicated verbally or in writing by such patients. In this case, management relied on objective physiologic markers - including heart rate, blood pressure, diaphoresis, and observed tremor - as well as collateral history from family members and caregivers. This aligns with recommendations from Felhofer et al. [8], who advocate for modified, nonverbal assessment algorithms incorporating vital sign trends and behavioral cues. Visual analog scales and pictogram-based tools have shown promise for pain and distress assessment in D/HH populations [5], though these remain unvalidated in the setting of alcohol withdrawal.

The absence of validated detoxification or addiction-screening instruments for D/HH patients represents a systemic gap in clinical practice [9,10]. The development and validation of sign-language and low-literacy-adapted tools should therefore be prioritized to promote equitable diagnostic accuracy and safety.

Communication barriers were the central determinant of treatment complexity in this case. The patient’s congenital deafness and mutism precluded both spoken and signed expression, while illiteracy rendered written communication futile. Effective engagement required a multimodal strategy combining interpreter mediation, nonverbal observation, and family-assisted contextual interpretation. Interpreters in such contexts must possess advanced skills, including fluency in ASL, the ability to adapt to non-standard or limited signing, and specialized training in mental health and addiction medicine to ensure accurate emotional and contextual translation. Prior studies emphasize that interpreters with these competencies are essential for bridging communication gaps and safeguarding the quality of psychiatric and addiction care [5,7].

Psychosocial intervention was carefully adapted to the patient’s communicative and cultural context. Interpreter‑facilitated therapy sessions allowed for some degree of affective expression, while family‑centered counseling targeted bereavement‑related triggers for relapse. In addition, therapeutic strategies emphasized nonverbal observation, structured routines, and simplified visual aids to compensate for limited literacy and expressive capacity. Interpreters required advanced skills in ASL, mental health terminology, and cultural competence to ensure accurate translation of emotional nuance and addiction‑related concepts [5]. Despite these adaptations, the patient’s communication limitations continued to hinder full therapeutic engagement and autonomy, reflecting systemic barriers faced by deaf and hard‑of‑hearing individuals in addiction treatment. These challenges underscore the need for specialized training of interpreters, integration of multimodal communication strategies, and development of accessible therapeutic frameworks, echoing broader concerns documented in the D/HH addiction literature [4,7,11].

The broader implications of this case extend beyond the individual patient and highlight the critical role of social determinants of health in shaping outcomes for deaf and hard‑of‑hearing (D/HH) individuals with substance use disorders [10,12]. Linguistic isolation was a central barrier, as the patient’s congenital deafness, mutism, and limited literacy precluded access to standard communication channels, thereby restricting his ability to engage fully with care. Cultural marginalization compounded these challenges, with limited recognition of deaf culture and inadequate tailoring of treatment approaches to his communicative needs. Structural barriers, including the shortage of certified sign‑language interpreters, lack of staff trained in deaf culture, and absence of accessible educational materials, further constrained his treatment options.

Socioeconomic determinants also played a role: the patient’s reliance on disability benefits and absence of vocational opportunities reflected systemic inequities in employment access for individuals with communication disabilities. Family support partially mitigated these barriers, but bereavement following the death of his sister intensified vulnerability to relapse. Collectively, these factors illustrate how social determinants - communication access, cultural competence, socioeconomic stability, and family support - directly influenced both the complexity of his treatment and the risk of poor outcomes.

The result, consistent with broader D/HH addiction literature [10,12,13], is a pattern of chronic under‑diagnosis, undertreatment, and relapse among deaf individuals with substance use disorders. Many treatment centers lack certified sign-language interpreters, staff trained in deaf culture, or accessible educational materials [13]. The result is chronic under-diagnosis, undertreatment, and relapse among D/HH populations with substance use disorders [2,8,13]. Addressing these inequities requires not only pharmacologic and psychosocial interventions but also systemic reforms: expanding interpreter services, training providers in deaf culture, developing accessible health education materials, and ensuring equitable vocational and social support.

To mitigate these disparities, addiction medicine should incorporate universal design principles, creating materials and screening tools that accommodate varying literacy and communication modalities from the outset. Policymakers should mandate interpreter availability and integrate telehealth-based e-therapy models, which have demonstrated success in bridging geographic and linguistic gaps [10]. Additionally, multidisciplinary rehabilitation teams - including addiction specialists, mental health providers, interpreters, and social workers experienced in disability care - are critical to promoting adherence and recovery.

## Conclusions

This case underscores the challenges of treating severe alcohol use disorder in a deaf, mute, and functionally illiterate patient, where standard withdrawal assessments were not feasible. Successful management required reliance on physiologic monitoring, collateral history, and interpreter‑facilitated communication.

Beyond the immediate medical concerns, the patient’s recovery was strongly influenced by social factors that shaped his ability to engage in care. His deafness and limited literacy created significant barriers to communication, making it difficult to access standard treatment approaches or benefit from written materials. The lack of certified sign‑language interpreters and providers familiar with deaf culture further restricted meaningful participation in therapy to impact long-term changes. Financial instability, reflected in his dependence on disability benefits and absence of vocational opportunities, added another layer of risk for relapse. Although family support offered some protection, the loss of his sister intensified feelings of grief and reduced his social safety net. Taken together, these circumstances highlight how communication access, cultural awareness, economic stability, and family involvement directly affect treatment complexity and outcomes for deaf individuals struggling with substance use disorders.

The case highlights the importance of culturally adapted assessment tools, early involvement of professional interpreters, and systematic attention to social determinants of health to optimize outcomes in this population.
